# Prevalence of *Infectious Spleen and Kidney Necrosis Virus* (ISKNV), Nervous Necrosis Virus (NNV) and Ectoparasites in Juvenile *Epinephelus* spp. Farmed in Aceh, Indonesia

**DOI:** 10.3390/pathogens9070578

**Published:** 2020-07-16

**Authors:** Bakhtiar Sah Putra, Paul M. Hick, Evelyn Hall, Richard J. Whittington, Razi Khairul, Evarianti, Nurbariah, Joy A. Becker

**Affiliations:** 1 Sydney School of Veterinary Science, The University of Sydney, Camden 2570, Australia; nicolas.bakhtiar@gmail.com (B.S.P.); paul.hick@sydney.edu.au (P.M.H.); evelyn.hall@sydney.edu.au (E.H.); richard.whittington@sydney.edu.au (R.J.W.); 2 Brackish Water Aquaculture Development Centre, Ujung Batee, Banda Aceh PO Box 46, Indonesia; Khairul.rz@gmail.com (R.K.); bollybolly150@gmail.com (E.); nur.chy14@gmail.com (N.); 3 School of Life and Environmental Sciences, The University of Sydney, Camden 2570, Australia

**Keywords:** *Megalocytivirus*, ISKNV, RSIV, nervous necrosis virus, *Betanodavirus*, ectoparasite, iridovirus, *Epinephelus* spp., biosecurity, viral encephalopathy and retinopathy

## Abstract

A cross-sectional survey was used to estimate the prevalence of infections with the *Infectious spleen and kidney necrosis virus* (ISKNV, *Megalocytivirus*), nervous necrosis virus (NNV, *Betanodavirus*), and infestations with ectoparasites during the rainy season in juvenile grouper (*Epinephelus* spp.) farmed in Aceh, Indonesia. The survey was intended to detect aquatic pathogens present at 10% prevalence with 95% confidence, assuming 100% sensitivity and specificity using a sample size of 30 for each diagnostic test. Eight populations of grouper from seven farms were sampled. Additional targeted sampling was conducted for populations experiencing high mortality. Infection with NNV was detected at all farms with seven of the eight populations being positive. The apparent prevalence for NNV ranged from 0% (95% CI: 0–12) to 73% (95% CI: 54–88). All of the fish tested from the targeted samples (Populations 9 and 10) were positive for NNV and all had vacuolation of the brain and retina consistent with viral nervous necrosis (VNN). Coinfections with ISKNV were detected in five populations, with the highest apparent prevalence being 13% (95% CI: 4–31%). *Trichodina* sp., *Cryptocaryon* *irritans* and *Gyrodactylus* sp. were detected at three farms, with 66% to 100% of fish being infested. Hybrid grouper sourced from a hatchery were 5.4 and 24.9 times more likely to have a NNV infection and a higher parasite load compared to orange-spotted grouper collected from the wild (*p* < 0.001). This study found that VNN remains a high-impact disease in grouper nurseries in Aceh, Indonesia.

## 1. Introduction

Grouper (subfamily Epinephelinae) aquaculture is a well-established industry in Asia with 155,000 tonnes produced annually. Behind China and Taiwan, Indonesia is the third largest producer with 17,000 tonnes per year (or 11% of global production) [1]. Grouper culture is an important economic activity in coastal areas throughout Indonesia, and provides livelihoods not only for those directly involved in farming activities, but also for those involved in other parts of the value chain, such as input supply and marketing [2,3,4].

In Indonesia, the production cycle for grouper (*Epinephelus* spp.) comprises three stages; hatchery, nursery and grow-out, which tend to be concentrated in geographically distinct areas across the island nation [1]. The hatchery stage begins with fertilised eggs harvested from broodstock tanks and ends when the larvae have metamorphosed and reached 2–3 cm total length (TL) [5]. This is followed by the nursing stage, which lasts between 30 and 50 days, where the fish are grown to 5–10 cm TL and then grow out in sea cages to market size (500–800 g) in about 9 months [6,7].

The nursery phase for grouper production can be undertaken either in shore-based tanks or in coastal ponds [7]. In ponds, fish are initially cultured in small net-pens (ranging in size from 1 to 2.5 m^3^) with a 1 mm mesh size at a density of 800 fish/m^3^ [6,7]. Generally, water quality in coastal ponds is managed by flushing twice each month during the highest tides [6,7]. For the first 14 days after transfer, the juvenile fish are fed on small wild fish and mysid shrimp that are cultured directly in the pond. Following this, fish are graded based on size and transferred to a similar size net-pen with a 4 mm mesh size at a density of 400 fish/m^3^ and fed a diet of chopped up low value “trash” fish harvested from the local estuary [1,6,7]. In Aceh, the two main species cultured are orange-spotted grouper (*Epinephelus coioides*), which are captured from the wild, and tiger grouper (*Epinephelus fuscoguttatus*), which are reared in hatcheries [6]. Novel species hybridizations have been successful for tiger grouper cross-bred with other grouper species, in particular the giant grouper (*Epinephelus lanceolatus*) [8,9,10]. Hybrid grouper grown in a net-pen culture system within an earthen pond were shown to have faster growth rates and improved disease resistance to pathogenic bacteria compared to juveniles derived directly from both parent species [10].

In a survey of pond-based grouper nurseries in Aceh, survival was generally observed to be approximately 75% but varied from 15% to 98% [6]. Anecdotally, farmers report low survival was associated with the rainy season when water temperature and salinity can drop quickly in the ponds due to heavy rainfall e.g., from approximately 31 °C to 27 °C and 33 ppt to 27 ppt, respectively [6].

Larval and juvenile grouper are susceptible to epidemic mortality outbreaks of disease caused by infection with *Betanodavirus*, nervous necrosis virus (NNV) [11] and by infections with *Megalocytivirus* [12]. Fish nodaviruses are known to infect more than 40 marine fish species and can cause the devastating disease known as viral nervous necrosis (VNN) (or viral encephalopathy and retinopathy) [11]. VNN is considered to be a serious disease worldwide; it is included in the OIE Manual of Diagnostic Tests for Aquatic Animals [13] and is regionally listed by the Network of Aquaculture Centres in Asia-Pacific [14]. Disease syndromes due to infections with *Megalocytivirus*, specifically red sea bream iridovirus (RSIV) and *Infectious spleen and kidney necrosis virus* (ISKNV), frequently occur in east and Southeast Asia [15] with more than 50 species of marine and freshwater fish considered to be susceptible [16]. Megalocytiviruses are subject to notification in many countries and infection with RSIV is listed by the World Organization for Animal Health [17] under a definition that includes infections with RSIV and the type species ISKNV. The international trade in juvenile fish for aquaculture and the ornamental pet industries has widened the geographical distribution of megalocytiviruses by translocating diseased live fish to Europe [18], United States [19], Australia [20,21], and Japan [22].

Grouper are known to exhibit prolonged sub-clinical infections with NNV and megalocytiviruses with disease manifesting under conditions involving various combinations of host and environmental risk factors [23,24]. Infestations with parasites with direct life cycles such as ciliates, monogeneans, and crustaceans cause mortality events in all stages of grouper aquaculture if left untreated [25,26]. There are no data on the prevalence of these pathogens in grouper farmed in Aceh. The objective of this study was to estimate the prevalence of infections of key viral pathogens (NNV and ISKNV), and to quantify the ectoparasite infestation in juvenile grouper held in coastal ponds during the rainy season in Aceh, Indonesia.

## 2. Results

**Figure 1:**
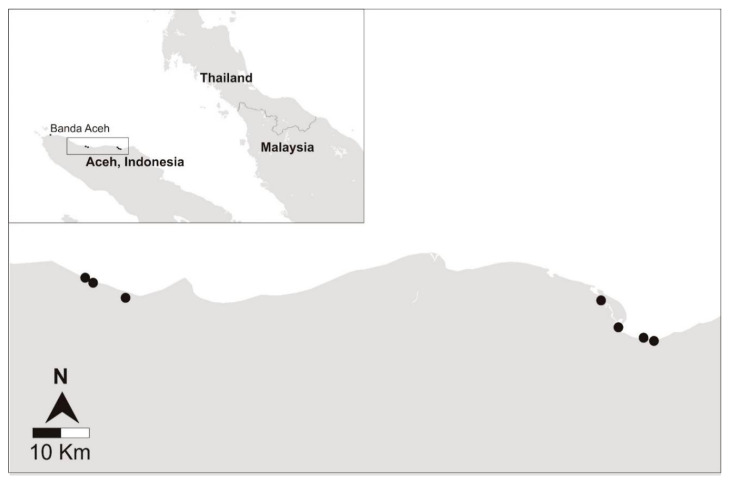
Sampling locations of the seven grouper nurseries in Aceh, Indonesia (inset) (Map generated using Mapbox ©).

Seven grouper farms were surveyed from four districts on the east coast of Aceh from 29 November to 4 December 2015 (Figure 1; Table 1). Eight populations of fish (Populations #1 to #8) were randomly sampled from these farms (Table 1). Targeted sampling of diseased fish was also completed on Populations #4 and #8 and referred to as Population #10 and #9, respectively (Table 1). Three grouper species, all belonging to the genus *Epinephelus* were sampled, including wild-sourced orange-spotted grouper (*Epinephelus coioides*) and hatchery-produced hybrids known as Cantang (*E. fuscoguttatus* ♀ × *E. lanceolatus* ♂) and Cantik (*E. fuscoguttatus* ♀ × *E. polyphekadion* ♂) (Table 1).

The necropsy identified that the fish had internal and external abnormalities, such as skin ulcers, changes in skin colour (dark or pale), gill pallor or haemorrhage, emaciation, unilateral or bilateral exophthalmia, cataract, short operculum, fin erosion and/or haemorrhage, ascites, changes in the size and the colour of the spleen, anterior kidney, posterior kidney and liver (Table 2; Table S1). The proportion of fish in Populations #1 to #8 with gross pathological signs ranged between 10% (95% CI: 4.7–18%) and 80% (95% CI: 70–88%) (Table 2). There were no significant associations between any gross abnormalities and infection with NNV (*p* = 0.876) and ISKNV (0.702). There was no significant association between a fish having any observed abnormality or skin/gill abnormality with the parasite load (*p* = 0.405 and *p* = 0.707). All populations of fish had gross pathological changes associated with the hematopoietic organs (e.g., liver, kidney and spleen) and abnormalities were frequently observed for the skin and gills (Table 2). The proportion of fish with gross abnormalities was significantly higher in the targeted samples from diseased populations compared to the representative selection of fish (71.1% ± 4.76 vs. 27.2% ± 1.65, all *p* < 0.001).

### 2.1. Detection of Nervous Necrosis Virus Infection

Infection with NNV was detected at all farms and in seven of the eight populations from the representative sampling (Table 3). The apparent prevalence of NNV ranged from 0% (95% CI: 0–12) to 73% (95% CI: 54–88) from the representative populations. The quantity of NNV was generally low, less than 10^3^ copies of the genome per mg tissue, in the eight populations sampled in the cross-sectional survey.

At Farm 2, NNV was not detected in the wild-caught orange-spotted grouper (Population 3) and no NNV-associated lesions were observed in the histopathological sections of these fish (*n* = 15). However, NNV was detected at Farm 2 in the hatchery-sourced Cantang (Population 2) with an apparent prevalence of 40% (95% CI: 23–59%) (Table 3). These two populations were reported to have been at Farm 2 for 20 and 30 days, respectively.

From the representative sample, hybrid grouper sourced from the hatchery were 5.4 times more likely to have NNV infection compared to orange-spotted grouper collected from the wild (*p* < 0.001). In other words, wild grouper had a 14% chance of being infected with NNV compared to hatchery-sourced fish with a 76% chance. NNV infection was not associated with farm factors including number of net-pens (*p* = 0.094), regional district (*p* = 0.138), and pond size (*p* = 0.711). NNV infection was not associated with fish factors including weight (*p* = 0.416), number of days at the farm (*p* = 0.831) and being classified as having no gross pathological changes (*p* = 0.536). Fish length was associated with NNV infection (*p* = 0.001). Fish less than 5 cm in total length had a 77% chance of infection compared to fish measuring 5–10 cm having a 25% chance and fish over 10 cm having a 41% chance.

**Figure 2:**
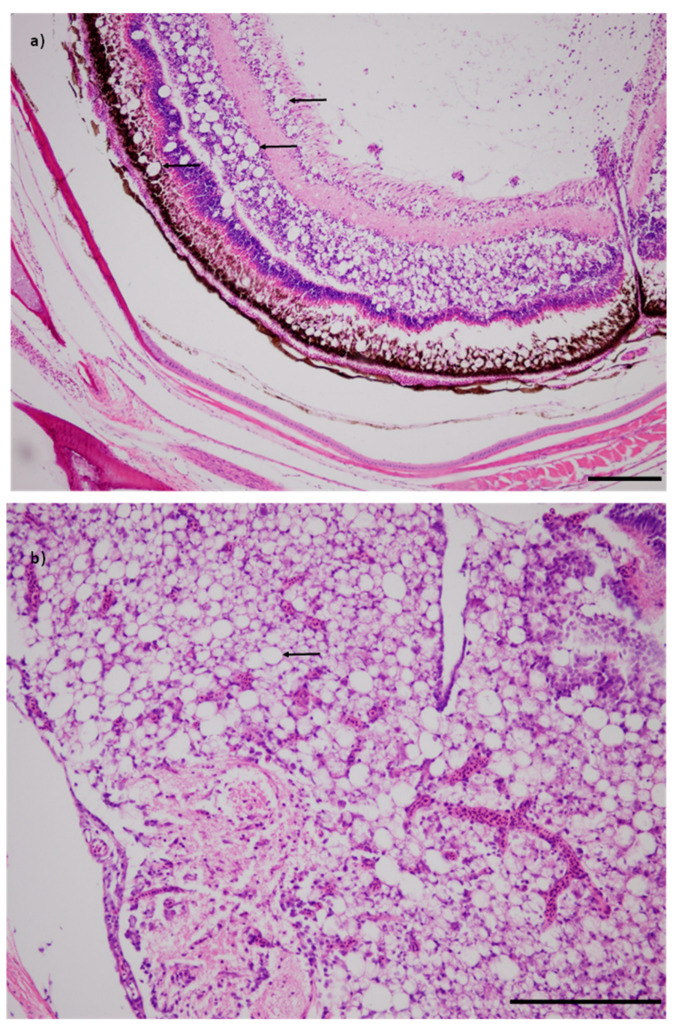
Juvenile grouper (*Epinephelus coioides*) with extensive vacuolation (arrows) in the retina (**a**) and brain (**b**) consistent with viral nervous necrosis that was associated with NNV infection (H&E stained, bar is 200 µm and 100 µm in (**a**) and (**b**), respectively).

All of the fish tested from the targeted samples of diseased fish (Populations 9 and 10) were positive for NNV and all fish had viral nervous necrosis disease-specific vacuolation of the brain and retina (Figure 2; Table 3). The apparent prevalence for the corresponding representative samples were 17% and 70% for Populations #8 and #4, respectively (Table 3). The NNV viral load among diseased fish approached 10^7^ copies of the genome per mg tissue, i.e, at least four orders of magnitude greater than in apparently healthy fish in the representative samples.

### 2.2. Detection of ISKNV Infection

Five of seven farms and five of eight of the representative populations were infected with ISKNV, with the highest apparent prevalence being 13% (95% CI: 4–31) (Table 3). Characteristic basophilic inclusion bodies were observed in the liver from one Cantik grouper from Farm 6 (Population #7) (Table 3). No microscopic lesions associated with ISKNV infection were found in any other population.

ISKNV infection was not associated with farm factors including number of net-pens (*p* = 0.912), regional district (*p* = 0.429), and pond size (0.561). Nor was ISKNV infection associated with fish factors including hatchery or wild source (*p* = 0.129), weight (*p* = 0.225), length (*p* = 0.992), number of days at the farm (*p* = 0.663) and being classified as having no observed gross pathological changes (*p* = 0.522). Both target populations (Population #9 and #10) were infected with ISKNV at a similar apparent prevalence to the representative sample of the same population (Population #8 and #4, respectively). The viral quantity for ISKNV was considered moderate to high (up to 3.0 × 10^8^ copies of the genome per mg tissue) (Table 3).

### 2.3. Parasitology

Parasite examinations were completed at three farms and two thirds to 100% of the fish had at least one ectoparasite (Table 4). Based on the ectoparasite sampling protocol used in this study, the mean abundance of ectoparasites per fish ranged from 5 to 72 (Table 4). The three parasites that were identified were putatively identified as the monogenan *Gyrodactylus* spp. and the ciliated protozoans *Trichodina* spp. and *Cryptocaryon irritans*. All three parasites were found at each farm with the exception that *C. irritans* was not found at Farm 6 (Table 4). The most common infestation was *Gyrodactylus* spp. followed by *Trichodina* spp., although the latter was the most abundant parasite (Table 4). Hatchery-sourced fish were 4.3 times more likely to have a diversity of parasite species than fish collected from the wild (*p* = 0.035). Hatchery fish were 24.9 times more likely to have a higher parasite load than wild-sourced fish (*p* < 0.001). The predicted mean ± standard error for hatchery- and wild-sourced grouper was 12.2 ± 1.2 parasites/g and 1.3 ± 0.88 parasites/g, respectively (*p* < 0.001). Length was not found to be significant in the multivariate model (*p* = 0.189).

## 3. Discussion

Disease has been identified as the primary constraint to the production of aquatic species, impeding both economic and social development in many developing countries [27,28,29]. The current study represented the first surveillance of NNV and ISKNV at grouper nurseries in Indonesia. The results showed that infections with NNV were evident at all seven farms and associated NNV disease was present at the time of the survey in two populations. Although disease caused by NNV is limited to larvae and young juveniles of many species [30], in the case of grouper, juvenile and adult fish can be affected [31,32]. The detection of low viral loads of NNV at a moderate apparent prevalence in the majority of the populations indicated that subclinical infection was occurring. This may be indicative of previous disease outbreaks. Alternatively, the presence of NNV might be indicative of ongoing outbreaks incorporating very low mortality rates and reduced production.

Disease-specific histopathological lesions associated with ISKNV infection were detected at one farm while infection was detected at five of seven farms. The disease in grouper caused by ISKNV infection has a range of presentations from acute disease outbreaks with 60–100% mortality, but also chronic disease with low grade mortality over a period of several months [16]. Whilst the latter was possibly detected in the present study, it is possible that temporally discrete disease outbreaks have impacted production on these farms.

There was a high prevalence of parasitic infestations and high parasite load at the three farms where parasite examination was possible. The parasite burden and diversity of species for hatchery-sourced grouper was significantly higher compared to wild-sourced fish. The pparasitic taxa identified were *Trichodina* spp., *Gyrodactylus* spp. and *Cryptocaryon irritans*, all of which are known to cause reduced growth and mortality in grouper aquaculture [26,33]. The results of this study suggest that disease syndromes with high prevalence and unknown aetiology, i.e., not associated with the detection of NNV and ISKNV, caused gross pathological changes to kidney, liver and spleen and skin ulceration.

Both NNV and ISKNV are cosmopolitan viral pathogens that can infect over 40 species of marine fish and are endemic in many parts of the world including grouper in Indonesia [11,12]. The present study confirms that NNV is a significant pathogen for grouper nursing in Aceh and should be a key focus for improved health management. Although the source of NNV and ISKNV remains unknown, there was a significantly greater likelihood of infection with either virus for fish sourced from a hatchery. A study in the South China Sea across 80 fish species found the same trend, with cage-reared fish (63%) significantly more likely to be infected with NNV compared to wild-caught fish (42%) [34]. In our study, only wild caught orange-spotted grouper were available at the farms, thus leading to an intrinsic linkage between species and source of fish. Differences in host species susceptibility to NNV infections is a gap in our current knowledge of NNV epidemiology.

Our study demonstrated that a high proportion of fish from each farm were showing a wide range of gross pathological changes that were independent of being infected with NNV and ISKNV or infested with ectoparasites. It is postulated that this has arisen from a combination of infectious, environmental and nutritional diseases. An integrated approach to disease management would reduce exposure to these pathogens through biosecurity, addressing risk factors for disease severity and vaccination for preventative measures. Relevant biosecurity measures would include batch culture, sourcing infection-free seed, appropriate disposal of dead and sick fish and reducing incursion of wild and trash fish. Expression of VNN and the impact of disease can vary with stress [11]. The occurrence of clinical and subclinical NNV infection concurrently in this study highlights the potential for improved farm management, nutrition and quality of seed to reduce the impact and severity of disease. A commercial vaccine is available for the RSIV genotype of *Megalocytivirus* and numerous vaccines have been described for NNV [26]. Vaccination is desirable in this circumstance as older fish remain susceptible to these key viral pathogens that are ubiquitous. Further, evaluation of the field efficacy and cost–benefit of vaccines for the purpose of improved productivity of grouper nursing is indicated.

In agreement with a survey on marine aquaculture in Vietnam [35], we observed that all farms had multiple species in culture and farmers reported similar management practices for acquiring fish, feeding, grading and separating size classes, and harvesting grouper (data not shown). In our study, only fingerlings of the same species and age were selected from a net-pen for testing. However, it was observed that grouper escape the net-pens during grading and harvest. As the ponds are not drained between batches of fish, large adult-sized grouper and other species of fish were observed in the ponds. There is potential for farm-level and district-level biosecurity measures to be implemented. For the farms visited, all fish, regardless of species and age, were held in several net-pens contained within one pond, even though numerous other production ponds were available. It was recommended to increase the number of ponds in production during the wet season and dry off any ponds not in use. This will serve to reduce the risk of horizontal spread of parasitic and viral pathogens by inhibiting the life cycles and by reducing the number of susceptible hosts with lower stocking densities. Fallowing ponds will allow for a complete harvest of aquatic animals and removal of any non-target species prior to re-stocking. The use of hatchery-produced hybrid grouper (*E. lanceolatus* × *E. fuscoguttatus*) is encouraged if they can be purchased certified free of NNV and ISKNV, as they have been shown to have improved growth rates and disease resistance to bacterial challenge compared to the their parent species [10]. Further, farmers reported that seawater was taken directly from the local estuary without filtration via a multi-user canal and dead fish were often disposed of in the canal. Filtration of in-coming seawater and off-site burial of moribund and dead fish were recommended changes to farm management.

Parasitic diseases have a major impact on global finfish aquaculture and in many regions they represent a key constraint to production, sustainability and economic viability [36]. We observed parasite infestation with more than two-thirds of fish having at least one parasite and an average of 5 to 72 parasites per fish. The parasite taxa observed all have direct life cycles and are considered generalists, in that they infest many fish hosts. Metazoan ectoparasites have been shown to aid in the spread of other aquatic pathogens as they have been shown to be mechanical vectors for viral pathogens [37]. The economic cost of on-going parasitic infections in aquaculture is difficult to calculate as it is often complicated by other disease outbreaks and management practices. (e.g., parasitic treatments) [36]. The development of a region-specific integrated parasite management plan is needed for the grouper nursing farms in Aceh. The management plan needs to consider the infection dynamics of these parasite species, the application of a broad range of management strategies (e.g., reducing reliance on trash fish as feed, restricting transmission from fomites) and direct treatment intervention (e.g., in feed medication or chemical bathing).

All of the farms in this study fed a low value ‘trash’ fish diet and it is considered a common management practice for this stage of grouper production in Indonesia [1]. This practice hinders the expansion of aquaculture and supports the spread of pathogens [35,38]. The feeding of trash fish has been shown to produce similar growth rates in tiger grouper (*E. fuscoguttatus*) compared to those fed commercial pellets, and there was no economic benefit to using pellets [38]. However, there was evidence of significantly reduced survival in grouper-fed trash fish [38], although the causes of poor survival were not investigated. Another study at Indonesian sea cages compared the parasite diversity and abundance in orange-spotted grouper (*E. coioides*) being fed trash fish or commercial pellets [33]. It was determined that endoparasites and parasites with direct life cycles (e.g., *Trichodina*) had higher prevalence and abundance in the group-fed trash fish [33]. The economic considerations of parasitic infestations need to be considered in the context of the regional farming practices.

This study showed that a major cause of disease in juvenile grouper during the rainy season was VNN caused by infectious with NNV. Additionally, co-infestations with ectoparasites and infections with ISKNV may have contributed to poor growth and survival. Hatchery-sourced fish had a significantly higher chance of being infected with NNV (5.4 times) and ectoparasites (24.9 times) compared to the wild-sourced grouper. In order to prevent disease outbreaks at the grouper nurseries, several regional-specific biosecurity recommendations could be implemented, in particular the requirement to stock fish that were certified disease-free.

## 4. Materials and Methods

### 4.1. Farm Selection

In July 2015, approximately four months prior to the rainy season, an informal in person discussion in Bahasa Indonesia was conducted with the owners at each of 24 grouper nurseries in Aceh, Indonesia. Farms were located along the coast of the Malacca Strait covering approximately 400 km between Sagoe (5°30′58.0″ N 95°47′42.1″ E) and Langsa (4°28′23.0″ N 97°58′32.5″ E). Seven grouper nurseries were selected for inclusion in the cross-sectional disease survey based on the availability of fish in the farm at the time of the study, willingness to participate, personal safety and accessibility to the laboratory within 24 h (Figure 1; Table 1). Sampling occurred between 28 November and 5 December 2015. Animal ethics approval was obtained from the University of Sydney Animal Ethics Committee (approval number: 2013/6027).

### 4.2. Sample Size and Fish Selection

A sample size of 30 fish per population was determined to provide 95% confidence of detecting a disease with a minimum expected prevalence of 10%, assuming 100% sensitivity and 100% specificity of the test [39]. In order to conduct molecular testing, histopathology and parasitology, this required a collection of 90 fish per population.

At each farm, a population of fish was defined as individuals of the same species and age. For all farms, each population of grouper was held in one pond but was distributed amongst several net-pens (hapas) (Table 1). When the population was contained within 3 to 6 net-pens, an equal number of fish were sampled from each. When the number of net-pens exceeded 6, an equal number of fish were sampled from 6 pens that were selected using a random numbers table. Further, an equal number of fish from each net-pen were randomly allocated to each diagnostic test.

To select the fish, the net-pen was pulled almost entirely out of the pond and tilted to one side to crowd the fish together. Fish were sampled using a scoop net and were collected into a bucket. The fish were selected by convenience without deliberate bias. This was the only method accepted by the farm managers that was suitable to minimize disruption to the health and production of the remaining fish. At two farms, the farm manager reported severe mortality in the population included in the cross-sectional survey. An additional targeted selection of moribund fish (15 fish per diagnostic test) showing clinical signs was completed and fish were dissected and sampled as described below. The clinical signs included abnormal swimming, skin lesions, loss of appetite and skeletal deformity.

### 4.3. Sample Collection

Necropsy was performed directly on site at the farms as the fish were collected. Fish were euthanized in pond water using 400 to 500 mg/L clove oil (diluted for use in a small amount of 70% ethanol). Fish were weighed, measured and examined for gross pathology. In order to avoid cross contamination, the workspace was disinfected using 200 ppm sodium hypochlorite then 70% ethanol, and equipment was disinfected using 200 ppm sodium hypochlorite for 10 min before rinsing in clean fresh water. A new sterile scalpel blade was used to necropsy each fish. Samples for PCR were preserved in 95% ethanol. For histopathology, tissues were fixed in 10% neutral buffered formalin and processed if needed to look for evidence of disease in populations of fish that tested positive for virus.

### 4.4. Parasitology

Parasitology was conducted at three nurseries where electricity was available to enable onsite microscopy. Examination for ectoparasites was conducted from a scraping of the mucus layer of the skin, a gill biopsy and a fin clip [40]. Briefly, both sides of the lateral body of the fish were scrapped from the posterior edge of the operculum to the base of the caudal fin using a sterile scalpel blade to collect the mucous on a glass slide. Then, a marginal portion of the left pectoral fin (approximately 0.5 cm × 1.5 cm) and a gill biopsy (approximately 10–15 primary lamellae) were placed on a glass slide with a few drops of pond water. These samples were covered with a glass coverslip and examined by an expert in marine parasitology (R. Khairul) immediately for parasites using a compound binocular microscope (Olympus CX21) for up to 10 min per fish. Fifteen specimens of each type of parasite from each farm were preserved in 70% ethanol for identification. At the diagnostic laboratory at the Brackish Water Aquaculture Development Centre, parasite morphology was used to assign a putative identification to the genus level.

### 4.5. Molecular Detection of Megalocytivirus and Nervous Necrosis Virus

#### 4.5.1. Tissue Homogenization and Nucleic Acid Purification

Tissues were homogenized using sterile disposable pestle grinders in phosphate buffered saline (PBS) at 1/10 *w/v* final dilution after air drying to remove ethanol. A pool of an equal portion of kidney, liver and spleen from one individual fish was prepared for *Megalocytivirus* tests and a pool of both retinas and brain to test for NNV. The homogenates were clarified by centrifugation for 10 min at 900× *g* and a 200 µL aliquot of the supernatant was used for nucleic acid purification using the High Pure Viral Nucleic Acid Kit (Roche, Penzberg, Germany) according to the manufacturer’s directions.

#### 4.5.2. RT-qPCR for Nervous Necrosis Virus

Real-time, reverse transcription PCR (RT-qPCR) was used to detect NNV according to previously described methods [41,42]. Samples were tested in duplicate using 5 µL of purified nucleic acid as template in 25 µL reactions prepared using the one-step qRT-PCR kit (Qiagen, Venlo, The Netherlands) on an ABI-7500 real-time PCR system (Applied Biosystems). Each plate included the following control reactions: positive control RNA containing the NNV genome, a negative extraction control, and a no-template RT-PCR negative control (nuclease-free water) (NTC1). The assay was run under the following conditions: 1 cycle of reverse transcription 45 °C 10 min, 1 cycle of hot start activation at 95 °C 10 min, 40 cycles of denaturation at 95 °C for 15 s, and annealing at 60 °C for 45 s. The threshold cycle (Ct) value for positive samples was defined as the fractional cycle number during which the ROX-normalized 6-FAM fluorescence signal exceeded the manual threshold of 0.05 dRN and provided a logarithmic amplification curve. A 6-step serial 10-fold dilution series containing 10^6^ to 10^1^ copies of linear plasmid DNA with NNV RNA2 sequence in nuclease-free water was amplified in duplicate on each plate for quantification of positive samples. A standard curve was prepared by plotting the log of the plasmid standard template quantity against the Ct value. This provided a measure of the quantity of NNV RNA, estimated as the number of copies of the NNV capsid protein gene per mg of tissue within the range of the standard curve, where the efficiency was between 90% and 110%.

#### 4.5.3. qPCR for *Megalocytivirus*

Quantitative PCR for detection of megalocytiviruses was performed according to the method described by Rimmer, et al. [43]. This assay will detect several viruses within the genus *Megalocytivirus*, including RSIV and the many genotypes of ISKNV. Briefly, the assay was performed in 25 µL reactions containing: 2.5 µL of template DNA; 250nM forward and reverse primers; 12.5 µL of Quantitech SYBR green master mix (Qiagen, Venlo, The Netherlands); and 7.25 µL molecular biology-grade nuclease-free water. Each plate included the following control reactions: positive control fish tissue infected with *Megalocytivirus*; negative control nuclease-free water (NTC 1) and nuclease-free water subjected to the nucleic acid purification process (NTC2 EXT). The PCR assay was performed using a 7500 real-time PCR system (Applied Biosystem) under the following conditions: 1 cycle of initial denaturation at 95 °C for 15 min; 40 cycles of denaturation at 95 °C for 30 s, annealing at 62 °C for 30 s, and extension at 72 °C for 30 s, with fluorescence acquisition at the end of the annealing step. The threshold cycle (Ct) values were determined with quantitative PCR software. Post amplification, a dissociation curve was determined by: dissociation of reaction products at 95 °C for 1 min; annealing at 55 °C for 30 s; heating to 95 °C at a rate of 1 °C every 30 s. Continuous collection of SYBR fluorescence signal indicated the temperature at which the reaction products dissociated. A single peak in the dissociation curve with a melting temperature of 85.0 °C ± 0.5 °C confirmed the specificity of the amplification product and was required to assign a positive result. A 6-step, serial 10-fold dilution series in nuclease-free water that contained 10^6^ to 10^1^ copies of the ISKNV major capsid protein gene sequence in linear plasmid DNA was amplified in duplicate for quantification of positive samples. This provided a measure of the number of genome copies per mg of tissue in the range of the standard curve that had an efficiency between 90% and 110%. Conventional PCR was used to amplify segments of the major capsid protein and ATPase genes as previously described (Rimmer et al. 2017). Purified amplification products were submitted to the Australian Genome Research Facility for Sanger sequencing. The ISKNV genotype was confirmed for at least one qPCR-positive fish from each population, as the number of bases different to the ISKNV reference sequence (GenBank Accession AF371960) was 1 or less.

### 4.6. Histopathology

Samples processed for histopathology included all samples from the two populations with disease (#9 and #10) and all fish with a positive qPCR for ISKNV from Populations 1 to 8. A random selection of 15 fish from Population 3 was included for comparison, as it had no positive detections for NNV and ISKNV. All other histopathology samples were held for future study.

The retinas, brain, liver, kidney and spleen were cut into 2 to 3 mm blocks and placed in one cassette for each fish. The tissues were processed by dehydrating through an ethanol concentration gradient and cleared using xylene, before embedding in a paraffin block. Sections were cut at 5 µm thicknesses, mounted onto a slide and stained with hematoxylin and eosin (H&E). The sections were examined by an experienced pathologist (R. Whittington) for up to 10 min per slide at up to 400× magnification. The microscopic examination was focused on observing specific lesions, such as vacuolation of the brain and retina consistent with viral nervous necrosis and megalocytic cells or inclusion bodies in each of the targeted organs. The megalocytic cells or inclusion bodies are defined as basophilic hypertrophic cells in the spleen, liver, heart, kidney, gills and other tissues [44].

### 4.7. Statistical Analysis

All statistical analyses were conducted in GenStat, v.18 (VSN International, Hemel Hempstead, UK). A *p* value < 0.05 was considered significant. For the apparent prevalence, exact binomial confidence intervals at the 95% level were calculated. For the regression analyses, categorization of continuous variables were as follows: days at farm (<30 days; 30 to 60 days; >61 days), number of hapas per pond (<5, 5 to 15; >15), and fish weight (<5 g; 6 to 9 g; 9 to 14 g; >14 g). Due to variation in population numbers, fish length was categorised differently for testing associations with viral pathogens (<5 cm, 5 to 10 cm and >10 cm) and parasitology testing (<7 cm; 7 to 9 cm; >9 cm). The gross pathology was categorized with 0 representing normal and changes in pathology coded from 1 to 3 representing changes in appearance (i.e., size and colour) (Supplemental Table S1).

The association between fish weight, fish length, percent of fish with any gross abnormality, gross abnormality in the hematopoietic organs (i.e., kidney, liver or spleen), gross abnormality in the skin and gills, or population (randomly selected vs. targeted) was assessed using Restricted Maximum Likelihood (REML) models. A Generalised Linear Mixed Model (GLMM) with an underlying binomial distribution was used to determine the effect of regional district, days at farm, pond size, number of net-pens, fish length, fish weight, species, source, hapa size, and gross pathology on presence of either ISKNV or NNV.

The parasite load was calculated as the total number of observed parasites divided by the fish body mass. The total number of parasites per fish and parasite load were coded to levels either 0, 1, or 2 corresponding with values 0, 1–10 and 10+ for number of parasites and 0, <1 or >2 for parasite load. Ordinal logistic regressions were conducted to determine the effect of fish source and length on categorised total number of parasites and parasite load. The effect of fish source, farm, fish length and weight and days at farm on the total number of parasites present and parasite load was investigated using REML models.

## Figures and Tables

**Table 1:** Summary of the populations of juvenile grouper (*Epinephelus* spp.) sampled at nurseries in Aceh, Indonesia.

Population ID	Farm ID	Regional District	Common Name	Days at Farm	Sample Size	Weight (g) ^1^	Total Length (cm) ^1^	Size of Ponds (ha)	No. of Net-Pens/Pond	No. of Net-Pens Sampled
1	1	Pidie Jaya	Orange-spotted	60	90	5.2 ± 1.7	7.1 ± 0.6	0.6	3	3
2	2	Pidie Jaya	Cantang	30	90	7.9 ± 2.7	7.7 ± 1.0	0.8	6	6
3	2	Pidie Jaya	Orange-spotted	20	90	5.6 ± 3.2	6.9 ± 1.5	0.8	14	6
4	3	Bireun	Orange-spotted	60	90	5.8 ± 6.2	6.4 ± 2.3	0.8	26	5
5	4	Aceh Utara	Cantik	7	90	1.3 ± 0.4	4.0 ± 0.4	0.25	36	5
6	5	Aceh Utara	Orange-spotted	75	90	5.0 ± 1.1	7.0 ± 0.6	2.5	3	3
7	6	Lhoksumawe	Cantik	32	90	6.7 ± 1.8	7.3 ± 0.6	1	5	5
8	7	Lhoksumawe	Orange-spotted	75	90	13.0 ± 8.6	9.4 ± 2.3	1.5	5	5
9 ^2^	7	Lhoksumawe	Orange-spotted	75	45	17.5 ± 17.9	10.4 ± 2.9	1.5	5	5
10 ^2^	3	Bireun	Orange-spotted	60	45	1.1 ± 0.3	4.9 ± 5.8	0.8	14	5

^1^: mean ± SD; ^2^: targeted sample of diseased fish.

**Table 2:** Summary of gross pathology observed in juvenile grouper (*Epinephelus* spp.) sampled at nurseries in Aceh, Indonesia.

Population ID	Farm ID	Common Name	Sample Size	Percent of Fish with Abnormalities	Percent of Fish with at Least One Observed Abnormality (%) ^1^					
					Liver Kidney Spleen	Gastro-Intestinal	Skin	Heart	Gill	Musculo-Skeletal ^2^
1	1	Orange-spotted	90	15.6	16.6	0	0	0	0	0
2	2	Cantang	90	34.4	31	15.5	2.2	0	0	2.2
3	2	Orange-spotted	90	25.6	25.5	5.5	0	0	1	0
4	3	Orange-spotted	90	80.0	80	17.7	0	0	37.7	0
5	4	Cantik	90	10.0	10	1	1	0	5.5	0
6	5	Orange-spotted	90	10.0	10	0	2.2	0	3.3	8.8
7	6	Cantik	90	17.8	17.7	0	2.2	0	16.6	1
8	7	Orange-spotted	90	23.3	22.2	0	14.4	2.2	13.3	11.1
9 ^3^	7	Orange-spotted	45	51.1	48.8	0	35.5	2.2	15.5	28.8
10 ^3^	3	Orange-spotted	45	91.1	88.8	4.4	37.7	0	48.8	2.2

^1^: see Supplemental Table S1 for descriptions of abnormalities; ^2^: includes scoliosis, lordosis or deformed operculum; ^3^: targeted sample of diseased fish.

**Table 3:** Apparent prevalence (AP) of nervous necrosis virus (NNV) and *Infectious spleen and kidney necrosis virus* (ISKNV) as detected by qPCR in juvenile grouper (*Epinephelus* spp.) sampled at nurseries in Indonesia.

Population ID	Farm ID	Common Name	*n*	Detection of NNV ^1^				Detection of ISKNV ^1^			
				AP (%)	95% CI	Quantity (Range)	Histo-Pathology ^2^	AP (%)	95% CI	Quantity (Range)	Histo- Pathology ^2^
1	1	Orange-spotted	30	13	4–31	BLOQ–9.8 × 10^2^	nd	0	0–12	0	nd
2	2	Cantang	30	40	23–59	BLOQ	nd	10	2–27	BLOQ	0/3
3	2	Orange-spotted	30	0	0–12	0	0/15	0	0–12	0	0/15
4	3	Orange-spotted	30	70	51–85	BLOQ–8.5 × 10^2^	nd	7	1–22	1.3 × 10^8^–1.8 × 10^8^	0/2
5	4	Cantik	30	73	54–88	BLOQ–8.5 × 10^2^	nd	0	0–12	0	nd
6	5	Orange-spotted	30	57	37–75	BLOQ	nd	3	0.1–17	BLOQ	0/1
7	6	Cantik	30	13	4–31	BLOQ	nd	13	4–31	BLOQ–2.9 × 10^7^	1/4
8	7	Orange-spotted	30	17	6–35	BLOQ	nd	13	4–31	BLOQ–3.0 × 10^8^	0/4
9 ^3^	7	Orange-spotted	15	100	78–100	BLOQ–5.9 × 10^6^	15/15	7	0.2–32	BLOQ	0/15
10 ^3^	3	Orange-spotted	15	100	78–100	1.2 × 10^6^–7.6 × 10^6^	15/15	7	0.2–32	BLOQ	0/15

^1^: BLOQ: Below the level of quantification of the qPCR assay, a high Ct value was obtained outside of the quantitative range; ^2^: not done; ^3^: targeted sample of diseased fish.

**Table 4:** Summary of ectoparasites observed on *Epinephelus* spp. at nurseries in Aceh, Indonesia. Ectoparasite observations were based on a mucus scrape on both sides of the lateral body, a gill biopsy (10–15 primary lamellae ca.) and a marginal portion of the left pectoral fin (approximately 0.5 cm × 1.5 cm).

Population ID	Farm ID	Proportion of Fish with Ectoparasites (%)	No. of Ectoparasites Per Fish (mean ± SD)	Parasite Genus	Apparent Prevalence (%)	Mean ± SD Number of Parasites Observed Per Fish		
						Mucus	Gill	Fin
6	5	20/30 (67)	5.0 ± 8.7	*Trichodina*	16.7	6.2 ± 8.8	22 ± 0	0
				*Cryptocaryon*	33.3	3.8 ± 1.8	3.8 ± 2.6	1 ± 0
				*Gyrodactylus*	50	1.7 ± 1.2	3.9 ± 4.2	0
7	6	30/30 (100)	72.2 ± 61	*Trichodina*	86.7	33.9 ± 24.1	22.1 ± 15.7	29.4 ± 23.8
				*Cryptocaryon*	0	0	0	0
				*Gyrodactylus*	76.7	0	23 ± 18	0
8	7	25/30 (83)	15.4 ± 61.0	*Trichodina*	43.3	8.4 ± 4.9	14.5 ± 17.4	8.6 ± 9
				*Cryptocaryon*	76.7	3.2 ± 1.5	3.3 ± 3.2	11 ± 0
				*Gyrodactylus*	33.3	0	8.4 ± 5.6	0
9 ^1^	7	15/15 (100)	40.4 ± 73.3	*Trichodina*	50	23.2 ± 20.1	34.7 ± 39.6	0
				*Cryptocaryon*	12.5	11 ± 0	0	0
				*Gyrodactylus*	93.4	3 ± 0	16.9 ± 30.8	0

^1^: targeted sample of diseased fish.

## Supplementary Materials

The following are available online at https://www.mdpi.com/2076-0817/9/7/578/s1, Table S1. Summary of the gross pathological changes observed and the scoring code for the statistical analysis. A score of ‘0’ was considered normal for all descriptors.
