# Supplementary material for: Prevalence of Infectious Spleen and Kidney Necrosis Virus (ISKNV), Nervous Necrosis Virus (NNV) and Ectoparasites in Juvenile Epinephelus spp. Farmed in Aceh, Indonesia
Source: Pathogens. 2020 Jul 16;9(7):578. doi: 10.3390/pathogens9070578 (PMC7400217; doi:10.3390/pathogens9070578)
Supplement: Supplementary file 1 [file pathogens-09-00578-s001.pdf]

Supplemental Table S1. Summary of the gross pathological changes observed and the scoring code for the statistical analysis. A score of '0' was considered normal for all descriptors.

**Description (scoring code)**	**Description (scoring code)**
Fin: frayed, eroded or haemorrhage (1)	Liver colour: dark (1); pale (2); two-tone (3)
Body surface: lesion or pale (1)	Liver size: reduced (1); enlarged (2)
Eyes: cataract or haemorrhage (1)	Spleen colour: dark (1); pale (2)
Gills: pale (1); haemorrhagic (2)	Spleen size: reduced (1); enlarged (2)
Body cavity: ascites (1)	Anterior kidney colour: pale (1)
Gall bladder: distended or over full (1)	Anterior kidney size: enlarged (1)
Swim bladder: distended (1)	Posterior kidney: dark (1); pale (2)
Adipose tissue: less 50% of organs covered (1)	Heart colour: dark (1); pale (2)
Intestine: abnormal, watery (1)	Heart size: enlarged (1)
Stomach: abnormal, watery (1)	
